# Clinical Pharmacology of 3,4-Methylenedioxymethamphetamine (MDMA, “Ecstasy”): The Influence of Gender and Genetics (CYP2D6, COMT, 5-HTT)

**DOI:** 10.1371/journal.pone.0047599

**Published:** 2012-10-24

**Authors:** Ricardo Pardo-Lozano, Magí Farré, Samanta Yubero-Lahoz, Brian O’Mathúna, Marta Torrens, Cristina Mustata, Clara Pérez-Mañá, Klaus Langohr, Elisabet Cuyàs, Marcel·lí Carbó, Rafael de la Torre

**Affiliations:** 1 Human Pharmacology and Clinical Neurosciences Research Group, Neuroscience Research Program, IMIM (Hospital del Mar Medical Research Institute), Parc de Salut Mar, Barcelona, Spain; 2 Universitat Autònoma de Barcelona (UAB), Barcelona, Spain; 3 Universitat Pompeu Fabra (CEXS-UPF), Barcelona, Spain; 4 Disorders by Use of Substances Research Group, Neuroscience Research Program, IMIM (Hospital del Mar Medical Research Institute), INAD-Hospital del Mar, Parc de Salut Mar, Barcelona, Spain; 5 Department of Statistics and Operational Research, Universitat Politècnica de Catalunya, Barcelona, Spain; 6 CIBER de Fisiopatología de la Obesidad y Nutrición (CB06/03), CIBEROBN, Santiago de Compostela, Spain; The Scripps Research Institute, United States of America

## Abstract

The synthetic psychostimulant MDMA (±3,4-methylenedioxymethamphetamine, ecstasy) acts as an indirect serotonin, dopamine, and norepinephrine agonist and as a mechanism-based inhibitor of the cytochrome P-450 2D6 (CYP2D6). It has been suggested that women are more sensitive to MDMA effects than men but no clinical experimental studies have satisfactorily evaluated the factors contributing to such observations. There are no studies evaluating the influence of genetic polymorphism on the pharmacokinetics (CYP2D6; catechol-O-methyltransferase, COMT) and pharmacological effects of MDMA (serotonin transporter, 5-HTT; COMT). This clinical study was designed to evaluate the pharmacokinetics and physiological and subjective effects of MDMA considering gender and the genetic polymorphisms of *CYP2D6*, *COMT,* and 5-HTT. A total of 27 (12 women) healthy, recreational users of ecstasy were included (all extensive metabolizers for CYP2D6). A single oral weight-adjusted dose of MDMA was administered (1.4 mg/kg, range 75–100 mg) which was similar to recreational doses. None of the women were taking oral contraceptives and the experimental session was performed during the early follicular phase of their menstrual cycle. Principal findings show that subjects reached similar MDMA plasma concentrations, and experienced similar positive effects, irrespective of gender or CYP2D6 (not taking into consideration poor or ultra-rapid metabolizers) or COMT genotypes. However, HMMA plasma concentrations were linked to *CYP2D6* genotype (higher with two functional alleles). Female subjects displayed more intense physiological (heart rate, and oral temperature) and negative effects (dizziness, sedation, depression, and psychotic symptoms). Genotypes of *COMT val158met* or *5-HTTLPR* with high functionality (val/val or l/*) determined greater cardiovascular effects, and with low functionality (met/* or *s/s*) negative subjective effects (dizziness, anxiety, sedation). In conclusion, the contribution of MDMA pharmacokinetics following 1.4 mg/kg MDMA to the gender differences observed in drug effects appears to be negligible or even null. In contrast, 5*-HTTLPR* and *COMT val158met* genotypes play a major role.

**Trial Registration:**

ClinicalTrials.gov NCT01447472

## Introduction

Ecstasy (±3,4-methylenedioxymethamphetamine, MDMA) is a synthetic psychostimulant derived from phenylethylamine and the third most widely consumed illegal drug in the world [1]. This increase in abuse has become a growing public health concern since MDMA can produce acute toxicity and fatal episodes [2]. In addition, its chronic consumption is associated with neurocognitive deficits and increased psychopathology prevalence [3]–[6]. Several studies suggest that women are more vulnerable to drug abuse than males [7]. In some Western countries, female users present higher rates of consumption and dependence on ecstasy than men [1] and MDMA has been shown to produce stronger effects in them [8], [9]. Differences in drug disposition may contribute to this observation; nevertheless, the only pharmacokinetic study published to date does not have sufficient sample size to address this issue [10].

It has also been postulated that the genetic polymorphisms of the serotonin transporter *(5-HTT),* catechol-O-methyltransferase (*COMT)*, and cytochrome P450 2D6 (*CYP2D6),* are implicated in MDMA pharmacology as they may modulate both its pharmacodynamics and pharmacokinetics in humans [11], [12]. However, these genotypes have not been examined in MDMA clinical experimental pharmacology studies.

MDMA acts as an indirect serotonin (5-HT), norepinephrine (NE), and dopamine (DA) agonist. Disposition of 5-HT and DA at the synaptic cleft is modulated by the 5-HTT and the COMT enzyme, respectively [13]. The most common genetic variation of 5-HTT is the gene-linked polymorphic region (*5-HTTLPR)* which presents two variants: the long (*l*) one which results in a higher serotonin transporter mRNA transcription, and the short (*s*) one which causes lower transcription [14]. The *COMT* gene displays a functional polymorphism at codon 158 producing a valine (*val*) to methionine (*met*) substitution (Val158Met, rs4680) resulting in three genotypes (*val/val, val/met*, and *met/met*). Individuals with the *met* allele have a lower enzyme activity which leads to higher levels of extracellular dopamine [15].

The main metabolic pathway of MDMA in humans is its O-demethylenation to 3,4-dihydroxymethamphetamine (HHMA) by the CYP2D6, followed by the O-methylation of HHMA to 4-hydroxy-3-methoxymethamphetamine (HMMA) by COMT. CYP2D6 displays a large genetic variability determined by the number of functional alleles (FA). In 70–80% of Caucasians the most prevalent phenotype of CYP2D6 is the extensive metabolizer (EM) [16]. The clinical implications derived from *CYP2D6* genetic polymorphisms are a large variability of MDMA plasma concentrations and, as a consequence, a difference in drug response, adverse effects [12], and the bioactivation of MDMA to putative neurotoxic species in humans [17]. MDMA is also a potent inhibitor of CYP2D6 through the formation of a metabolic inhibitory complex [18]–[21]. This is of relevance since MDMA can temporarily (approximately ten days) convert EM subjects to apparently poor metabolizers (PM) [22], [23] thus increasing the risk of toxicity of other substances whose disposition is regulated by CYP2D6 [16].

Since the contributing factors for the observation of gender differences in MDMA induced effects have not been satisfactorily determined, and that there are no previously published reports about the influence of *CYP2D6, COMT val158met*, and *5-HTTLPR* genotypes on its pharmacokinetics and pharmacodynamics, the present study will examine both the impact of gender and genetics on the clinical pharmacology of MDMA.

## Materials and Methods

### Participants

Subjects were recruited by word of mouth and gave their written informed consent before inclusion in the study. Inclusion criteria were: healthy male and female adults, the recreational use of MDMA on at least ten occasions (two in the previous year), and the EM phenotype for CYP2D6 activity determined using dextromethorphan as a selective probe drug [23]. Women had to present a regular menstrual cycle and not be taking oral contraceptives. Exclusion criteria included daily consumption >20 cigarettes and >4 standard units of ethanol in men (>2 in women), and regular ingestion of medication in the month preceding the study. Eligible subjects underwent a psychiatric interview (Psychiatric Research Interview for Substance and Mental Disorders –PRISM) [24] to exclude the presence of major psychiatric disorders, history of abuse or drug dependence (except for nicotine dependence), and psychiatric adverse reactions after MDMA consumption. To confirm health status, volunteers were interviewed by a physician and underwent a general physical examination, routine laboratory tests, urinalysis, and a 12-lead electrocardiogram (ECG). All subjects were informed about the possible adverse effects during the study and were financially compensated for any inconvenience derived from their participation.

### Test Procedure

The design was open as the primary outcomes measured (pharmacokinetics) were objective. Subjects participated as outpatients, women during early follicular phase, in a session that began at 7∶30 a.m. in fasting conditions. Participants were requested to refrain from consuming any illicit drug of abuse for 2 weeks before the experimental session and were asked to follow a xanthine-free diet and alcohol-free ingestion 48 and 24 hours, respectively, prior to the beginning of each session. Before drug administration, urine samples were collected for a drug screen (opiates, cocaine metabolite, amphetamine, methamphetamine, cannabinoids, and phencyclidine) by a quick on-site test (Instant-View; ASD Inc., Poway, California, USA), and for pregnancy testing in females.

Negative results were mandatory for participation in the session. Single doses of medication for symptomatic treatment (e.g. acetaminophen for headache) were accepted up to the week preceding the trial. An indwelling catheter was inserted into a subcutaneous vein in the forearm of the non-dominant arm to obtain blood samples. Thereafter, the subjects remained seated in a quiet room. Drug administration commenced at 8∶30 a.m. with 250 mL of water, and a light breakfast was provided 2 h later. Tobacco smoking was permitted after lunch (6 h after MDMA administration). A psychiatrist evaluated volunteers 10 hours after MDMA administration. As the experimental session lasted 25 hours, subjects were admitted to the Clinical Research Unit for the night. Adverse events and concomitant medications were recorded.

### Drugs

Doses of MDMA were chosen according to data from previous studies [25], [21] and were within the range of the recreational doses reported for a single tablet of ecstasy. For the security of the volunteers, mainly the women, a 1.4 mg/kg of MDMA was administered (range, 75–100 mg). (R,S)-MDMA was supplied by the Spanish Ministry of Health and prepared in white, opaque, soft gelatin capsules by the Pharmacy Department of the Hospital del Mar (Barcelona, Spain).

### Pharmacokinetic Measurements

For determination of MDMA and metabolites, blood samples (8 mL) were collected at pre-dose, and at 0.33, 0.66, 1, 1.5, 2, 4, 6, 8, 10, 12, 25, and 48 hours after MDMA administration. Blood was collected in heparinized tubes and then centrifuged at 4°C for 10 min. The resulting four 1-mL plasma aliquots were stored at −20°C until analysis. MDMA, HMMA MDA (3,4-methylenedioxyamphetamine), and HMA (3-methoxy-4-hydroxyamphetamine) were analyzed following a previously reported method based on solid-liquid extraction and gas chromatography–mass spectrometry (GC/MS) [26].

### Genotyping

Genomic DNA was extracted from the peripheral blood leukocytes of participants using the Flexi Gene DNA kit (Qiagen Iberia, S.L., Spain). The *COMT* val108/158met and 5*-HTTLPR* genotyping was performed using polymerase chain reaction (PCR) as previously described [27]. The *CYP2D6* genotypes were determined using the PHARMAchip™ DNA array (Progenika Biopharma, Derio, Spain) [28]. Subjects were split according to genotype and associated functionality (CYP2D6, carrying 1 or 2 FA; *COMT val158met*, carrying the *val/val* or met/* alleles; and *5-HTTLPR,* carrying the l/* or *s/s* alleles) in order to have equilibrated groups for comparisons, considering genotypes and genders separately.

### Physiological Measures

Readings of non-invasive systolic blood pressure (SBP), diastolic blood pressure (DBP), heart rate (HR), oral temperature (OT), pupil diameter (PD), and esophoria (ESO) were taken 15 minutes prior to drug administration, at baseline (time 0), and at 0.33, 0.66, 1, 1.5, 2, 3, 4, 5, 6, 8, 10, 12, and 24 hours after drug administration. SBP, DBP, HR, and OT were recorded using a Carescape™ V100 monitor (GE Healthcare. Milwaukee, WI). Pupil diameter was calculated using a pupil gauge (Haab scale). The Maddox-wing device (AM, Clement Clark, London, U.K) was used to measure the balance of extraocular muscles and quantify exophoria and esophoria (MDMA effect) [25]. For safety reasons, ECG was continuously monitored during the first 12 h with a Dash® 3000 patient monitor (GE Healthcare).

### Rating Scales of Subjective Effects

Subjective effects were measured using a set of visual analogue scales (VAS), the Addiction Research Center Inventory (ARCI), and the Evaluation of the Subjective Effects of Substances with Abuse Potential (VESSPA) questionnaire at baseline and at 0.33*, 0.66*, 1, 1.5*, 2, 3, 4, 5, 6, 8, 10, 12, and 24 h (* only VAS) after MDMA administration. ***VAS.*** Twenty one 100-mm VAS labeled with different adjectives marked at opposite ends with “not at all” and “extremely” were administered [29]. ***ARCI.*** A Spanish validated version of a 49-item short form of ARCI was used [29], [30]. ***VESSPA*** is a validated questionnaire measuring MDMA-induced changes in subjective variables [30].

### Ethics

The study was approved by the local Ethics Committee (CEIC-Parc de Salut Mar), authorized by the Spanish Medicine Agency (AEMPS n°. 04–0013) of the Spanish Ministry of Health, and was conducted in accordance with the Declaration of Helsinki (Edinburgh, 2000) and good clinical practices. The study was registered at ClinicalTrials.gov (NCT01447472). Subjects gave their written informed consent before inclusion in the study.

### Statistical Analyses

A description of both male and female volunteers is given with means, standard deviations, and ranges (min and max values). Values of the peak plasma concentration (*C*_max_) of MDMA and its metabolites, and the time to reach Cmax (T_max_), were obtained directly from the plasma concentration-time profiles of MDMA, MDA, HHMA, and HMA. Area under the concentration-time curve values (AUC) was calculated using the trapezoidal rule. Absorption and elimination rate constant (K_a_ and K_e_, respectively) were estimated by log-linear regression of initial and four terminal data points. Values from physiological measures and subjective variables were transformed to differences from baseline and their peak effect (E_max_), T_max_ and AUC were calculated from effect-time profiles. The χ^2^-square test was used to check for the Hardy-Weinberg equilibrium.

To study gender differences with respect to pharmacokinetic (C_max_, AUC, T_max,_ T_1/2_, ratio MDMA/HMMA), physiological, and subjective effect parameters (E_max_, AUC, T_max_), ANCOVA models were used including both gender and weight-adjusted dose as independent variables. In addition, the possible effect of the *COMT val158met* and *CYP2D6* genotypes on the plasma concentration of MDMA, MDA, HHMA, and HMA was analyzed using separate ANCOVA models for genotypes as well as gender and weight-adjusted dose as further independent variables. Concerning pharmacodynamic variables, separate ANCOVA models, including gender and weight-adjusted dose, were applied to study the role of *COMT val158met* and *5-HTTLPR,* respectively. Due to the small sample size, instead of including the respective interaction terms in the ANCOVA models, separate nonparametric Wilcoxon tests comparing males with females were carried out for each genotype. Differences were considered to be significant if *p*<0.05. Statistical analyses were performed with the statistical software packages SPSS 12.0 for Windows (SPSS, Inc., 2003) and R, version 2.13.1 (The R Foundation for Statistical Computing). Data resulting from the ANCOVA model are presented with the *p* value and the 95% confidence interval of the mean difference between genders or genotypes.

## Results

### Sample Characteristics and Doses

A total of 45 Caucasian subjects [26 women (W), 19 men (M)] were recruited, but only 27 (12 W, 15 M) were included and completed the study. The reasons for the volunteers’ exclusion (n = 18) were: 12 W (10 having previous mental disorders [6 abuse of/dependence on drugs of abuse; 4 affective disorders]), 1 CYP2D6 PM phenotype, and 1 due to a positive drug screen test for cannabis and cocaine); and 3 M (2 CYP2D6 PM phenotype, and 1 with criteria of drug dependence). There were 3 withdrawals (2 W, 1 M) before the experimental session due to personal reasons.

The subjects included were tobacco smokers (10 W, 11 M), and had previous experience using alcohol (all 27 subjects), cannabis (8 W, 14 M), cocaine (6 W, 13 M), and gammahydroxybutirate (GHB, 4 M). In Table 1 anthropometric data and the oral and weight-adjusted MDMA doses are summarized according to gender and genotypes**.** Weight, height, and total MDMA dose administered were higher in men than in women, but body mass index was similar in both genders. According to genotypes, few differences were observed in MDMA doses in the distribution of gender and anthropometric data among the subgroups, age, however, varied in the *5-HTTLPR* genotype subgroups. Gender, genotypes, and the MDMA doses administered for each participant are specified in Table 2. The *COMT* (*p* = 0.44) but not the *5-HTTLPR* (*p* = 0.018) genotype fulfill the Hardy-Weinberg equilibrium.

**Table 1:** Anthropometric data of included volunteers and MDMA doses according to gender and genotypes (mean ± SD [min, max]).

Variable	Total dose *p.o*(in mg)	Weight-adjusted dose (in mg/kg)	Age (in years)	Weight (in Kg)	Height (in cm)	BMI (in kg/m^2^)
***Gender***						
Men (n = 15)	95.3±7.4** [80, 100]	1.36±0.1 [1.10, 1.51]	25.8±3.8 [19], [33]	70.8±10.8**[54.2, 91.2]	181.0±8.0** [170.5, 195.5]	21.6±3.2 [17.2, 28.7]
Wome (n = 12)	79.5±5.4 [75, 90]	1.42±0.05 [1.35, 1.50]	26.9±3.7 [21], [35]	56.0±4.7[50, 65.5]	166.7±4.8[157.3, 173]	20.2±1.5 [17.5, 23.1]
***Genotype***						
CYP2D6 - 1FA (n = 8)	88,8±10,9 [75, 100]	1,39±0,0 [1.27,1.45]	27.6±4.3 [22], [35]	64.2±9.4[52.9, 78.6]	175.1±7.8 [163.5, 184.0]	20.8±1.7 [19, 23.6]
CYP2D6–2FA (n = 19)	88,2±10,3 [75, 100]	1,39±0,1 [1.10, 1.51]	25.7±3.4 [19], [31]	64.3±12.3[50, 91.2]	174.5±10.8 [157.3, 195.5]	21.1±3.0 [17.2, 28.7]
COMT - *val/val* (n = 8)	84,4±10,8 [75, 100]	1,39±0,0 [1.20, 1.50]	26.0±4.2 [22], [35]	61.1±11.0[50, 83.5]	170.5±9.5 [157.3, 190.0]	20.8±1.6 [19, 23.1]
COMT - met/* (n = 18)	90,8±9,4 [75, 100]	1,38±0,1 [1.10, 1.51]	26.3±3.7 [19], [33]	66.4±11.4[50, 91.2]	177.1±9.5 [161.2, 195.5]	21.2±3.1 [17.2, 28.7]
5-HTTLPR - l/* (n = 18)	88,9±10,5 [75, 100]	1,39±0,1 [1.12, 1.51]	27.6±3.2* [22], [35]	64.7±11.3[50, 89]	174.9±10.6 [157.3, 195.5]	21.1±2.5 [17.2, 28.7]
5-HTTLPR - s/s (n = 9)	87,2±9,3 [75, 100,]	1,39±0,1 [1.10, 1.50]	23.8±3.6 [19], [30]	63.6±12.1[50, 91.2]	174.2±8.8 [164.5, 194]	20.8±3.1 [17.5, 27.6]

MDMA, ±3,4-methylenedioxymethamphetamine; CYP2D6, cytochrome P450 2D6; FA: functional alleles COMT, catechol-O-methyltransferase; 5-HTTLPR, gene-linked polymorphic region. BMI: body mass index; p.o: per os. *p<0.05; **p<0.01.

**Table 2:** MDMA doses (total oral and weight-adjusted) and genetic polymorphism of *CYP2D6, COMT,* and *5-HTTLPR* in women and men.

Female subjects	MDMA Dose(mg - mg/kg)	*CYP2D6* Genotype[FA]	*COMT* Genotype	*5-HTTLPR* Genotype	Malesubjects	MDMA Dose(mg - mg/kg)	*CYP2D6* Genotype [FA]	*COMT* Genotype	*5-HTTLPR* Genotype
**1**	80 - 1.38	*2/*10 [2]	*met/met*	*s/s*	**1**	100 - 1.20	*1/*1 [2]	*val/val*	*l/l*
**2**	75 - 1.50	*1/*2 [2]	*val/met*	*s/s*	**2**	100 - 1.12	*1/*17 [2]	*met/met*	*l/s*
**3**	75 - 1.42	*1/*3 [1]	*val/val*	*l/l*	**3**	100 - 1.36	*1/*4 [1]	*val/met*	*l/s*
**4**	75 - 1.41	*1/*4 [1]	*val/val*	*l/l*	**4**	90 - 1.37	*1/*4 [1]	*val/val*	*l/s*
**5**	75 - 1.50	*1/*35 [2]	*val/val*	*l/s*	**5**	80 - 1.48	*1/*10 [2]	*val/met*	*s/s*
**6**	90 - 1.45	*1/*1 [2]	*met/met*	*l/l*	**6**	100 - 1.45	*1/*2 [2]	*met/met*	*s/s*
**7**	80 - 1.39	*1/*1 [2]	*val/val*	*s/s*	**7**	100 - 1.10	*1/*9 [2]	*val/met*	*s/s*
**8**	80 - 1.48	*2/*35 [2]	*val/met*	*l/l*	**8**	90 - 1.51	*2/*41 [2]	*val/met*	*l/l*
**9**	80 - 1.38	*4/*41 [1]	*val/val*	*l/l*	**9**	100 - 1.37	*1/*2 [2]	*met/met*	*l/l*
**10**	75 - 1.42	*1/*41 [2]	n.a	*l/l*	**10**	100 - 1.27	*9/*10 [1]	*val/met*	*l/s*
**11**	90 - 1.37	*1/*1 [2]	*met/met*	*s/s*	**11**	100 - 1.44	*2/*4 [1]	*val/met*	*l/s*
**12**	80 - 1.35	*1/*2 [2]	*met/met*	*s/s*	**12**	100 - 1.49	*1/*2 [2]	*met/met*	*l/l*
					**13**	100 - 1.47	*1/*2 [2]	*val/val*	*s/s*
					**14**	80 - 1.36	*1/*2 [2]	*val/met*	*l/s*
					**15**	90 - 1.45	*1/*5 [1]	*val/met*	*l/l*

MDMA, ±3,4-methylenedioxymethamphetamine; CYP2D6, cytochrome P450 2D6; COMT, catechol-O-methyltransferase; val, valine; met, methionine; 5-HTTLPR,

gene-linked polymorphic region; FA: number of functional alleles. n.a: not available.

Gender, genotypes, and the MDMA doses administered for each participant are specified in Table 2.

Due to technical problems during analysis and/or not enough sample volume for complete analysis, the data available from pharmacokinetics and *COMT* genotype were for fewer subjects than for pharmacodynamics (Tables 3, 3b, 4, 5).

**Table 3:** Gender differences in pharmacokinetic parameters of MDMA and its metabolites (mean ± SD; for MDMA and HMMA: women n = 11 *vs.* men n = 15; for MDA and HMA: women n = 11 *vs.* men n = 12).

	AUC _0–25__h_ (µg·h·L^−1^)	C_max_ (µg/L)	T_max_(h)	T _1/2_ (h)	K_e_ (h^−1^)	K_a_ (h^−1^)	V_d_ (L)	Cl (L/h/kg)
**MDMA**								
Women	2667.1±616.8	190.3±60.7	2.6±1.4	11.0±12.6	0.10±0.05	2.22±1.42	375±178	20.8±6.0
Men	2212.5±649.7	187.5±38.9	2.8±1.0	7.4±2.9	0.11±0.04	2.79±0.84	478±237	34.5±12.0**
**HMMA**								
Women	2460.4±1681.6	170.2±131.5	3.6±2.8	15.5±9.9	0.05±0.18	n.d	n.d	n.d
Men	2463.7±1081.5	190.1±76.6	2.6±1.0	10.6±10.9	0.13±0.17	n.d	n.d	n.d
**MDA**								
Women	290.4±56.0	14.0±3.6	7.8±2.2	17.6±10. 1	0.02±0.13	n.d	n.d	n.d
Men	278.4±61.5	13.3±3.4	6.3±3.3	11.9±57.9	0.01±0.05	n.d	n.d	n.d
**HMA**								
Women	151.8±80.3	6.2±3.2	8.1±3.5	n.d ^a^	n.d ^a^	n.d	n.d	n.d
Men	140.2±65.9	6.0±2.5	7.5±2.7	n.d ^a^	n.d ^a^	n.d	n.d	n.d

MDMA, ±3,4-methylenedioxymethamphetamine; MDA, 3,4-methylenedioxyamphetamine; HMA, 3-methoxy-4-hydroxyamphetamine; HMMA, 3-methoxy-4-hydroxymethamphetamine; AUC: area under the concentration-time curve, C_max_: peak plasma concentration; T_max_: time with peak plasma; T_1/2_: half-life of elimination; K_e_: elimination constant; K_a_: absorption constant; Vd: apparent volume of distribution; Cl: clearance; n.d: not determined, due to high variability (a).

** *p*<0.01.

**Table 4:** Gender differences in physiological and subjective effects after MDMA administration (mean ± SD; women n = 12 *vs.* men n = 15) (only significant effects included).

Outcomes	AUC _0–4__h_ (Units)		AUC _0–6__h_ (Units)		AUC _0–24 h_ (Units)		E_max_ (Units)	
	Women	Men	Women	Men	Women	Men	Women	Men
**Physiological measures**								
Heart rate	52.5±16.0**	33.1±28.5	69.3±21.8*	41.7±42.7	118.5±81.0	62.2±145.6	26.2±8.3**	15.1±13.2
Oral temperature	1.2±0.8*	0.5±0.8	2.3±1.4*	1.1±1.1	6.4±5.7	3.7±3.5	0.6±0.4	0.4±0.5
**Subjective effects**								
VAS – Dizziness	20.4±17.0**	4.9±8.3	21.6±17.6**	5.2±8.9	22.7±18.4*	6.49±12.1	17.7±13.5*	6.1±9.6
VAS – Depression/Sadness	0.7±0.9	0.1±0.4	1.2±1.6*	0.1±0.4	2.2±4.9	0.18±0.4	0.9±1.3*	0.1±0.3
ARCI – PCAG (Sedation)	6.5±9.0	0.4±8.5	9.2±12.0	1.1±10.1	30.5±37.2*	1.8±10.5	4.5±3.7*	0.4±4.2
VESSPA – Sedation	17.0±12.2*	8.3±8.7	22.2±15.0*	10.1±10.3	54.4±55.3**	10.7±11.2	9.1±4.5**	3.9±3.6
VESSPA - Psychotic symptoms	6.6±4.0	4.1±5.6	7.8±4.5	4.4±6.0	9.2±6.7*	4.4±6.0	3.6±2.4	2.0±2.6

AUC: area under the effect-time curve; Emax: peak effect; Tmax: time of peak effect. VAS: visual analogue scale. ARCI: Addiction Research Center Inventory, PCAG: pentobarbital-chlorpromazine-alcohol group. VESSPA: Evaluation of the Subjective Effects of Substances with Abuse Potential.

* p<0.05,

** p<0.01.

**Table 5:** Genetic differences in physiological and subjective effects after MDMA administration (mean ± SD; women n = 12 vs. men n = 15) (only significant effects included).

Genotype	AUC _0-4__h_ (Units)		AUC _0–6__h_ (Units)		AUC _0–24 h_ (Units)		E_max_ (Units)	
*COMT val158met*	*val/val*	*met/* *	*val/val*	*met/* *	*val/val*	*met/* *	*val/val*	*met/* *
SBP	76.8±17.9*	47.3±23.9	100.4±29.5*	60.1±32.6	251±128.8**	73.4±115.6	34.2±7.0*	24.4±8.0
DBP	34.8±17.0	22.0±18.4	44.4±24.8	25.7±25.8	101±107.2*	12.4±69.8	19.2±6.5	14.4±5.6
Dizziness	5.6±9.3	13.2±15.7*	6.5±11.6	13.9±16.2*	7.1±13.3	15.3±17.8*	11.7±11.5	18.0±12.7*
VESSPA – Anxiety	17.5±6.4	23.0±12.9	19.6±7.7	27.3±14.0	21.1±9.1	30.7±16.9*	9.2±4.7	10.4±4.8
***5-HTTLPR***	*l/* *	*s/s*	*l/* *	*s/s*	*l/* *	*s/s*	*l/* *	*s/s*
SBP	65.4±24.0**	39.9±20.2	85.4±34.3**	48.8±26.0	173±153.4*	54.0±80.9	30.7±8.1**	21.4±6.6
DBP	30.4±18.0	17.5±16.5	37.8±24.5	19.1±25.5	60.0±90.0	4.6±81.6	17.4±6.2**	12.6±4.4
Heart rate	46.1±24.9	33.0±25.3	62.7±35.6*	36.6±35.9	121.9±94.8*	17.8±146.6	22.0±9.4	20.7±10.5
VESSPA – Sedation	8.8±9.2	18.8±12.1*	11.9±11.7	22.6±15.6	30.1±47.1	30.1±35.9	5.9±4.8	8.2±4.0

AUC: area under the effect-time curve; Emax: peak effect; COMT, catechol-O-methyltransferase; SBP: systolic blood pressure; DBP: diastolic blood pressure. 5-HTTLPR, gene-linked polymorphic region VESSPA: Evaluation of the Subjective Effects of Substances with Abuse Potential.

* p<0.05;

** p<0.01. N = [COMT, valval n = 8 vs. met/* n = 18; 5-HTTLPR, l/* n = 18 vs. s/s n = 9.

### Pharmacokinetics

#### Gender

**Figure 1:**
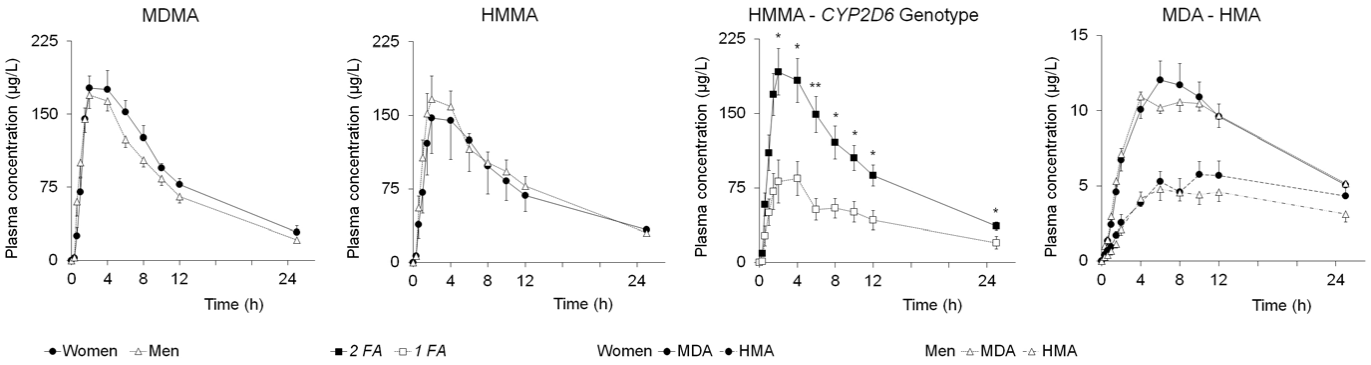
Plasma Concentrations of MDMA, HMMA, MDA, and HMA in both genders (mean ± standard error of the mean, SEM; for MDMA and HMMA: women n = 11 *vs.* men n = 15; for MDA and HMA: women n = 11 *vs.* men n = 12). Influence of *CYP2D6* genotype in plasma concentrations of HMMA (mean ± SEM; subjects with 2 FA n = 18 *vs.* with 1 FA n = 8. *p<0.05, **p<0.01.

No major gender differences were found for most of the pharmacokinetic parameters evaluated for MDMA and its metabolites (Table 3 and Figure 1). The only significant differences observed concerned MDMA plasma clearance (Cl) and the MDMA vs. HMMA AUC_0–12__h_ ratio. Men presented a higher Cl compared to women (*p* = 0.001, 95% CI: 6.13–21.08); and women displayed a higher MDMA vs. HMMA AUC_0–12__h_ ratio (*p = *0.027, 95% CI: 0.14–2.12) than men.

#### *CYP2D6* genotype

Subjects who were carriers of 2 FA of CYP2D6 presented higher mean values in HMMA plasma concentrations compared to volunteers with 1 FA [C_max_ (*p = *0.003, 95% CI = 47.6–199.8), AUC_0–4__h_ (*p = *0.008, 95% CI = 96.7–566.4) and AUC_0–25__h_ (*p = *0.003, 95% CI = 479.0–2148.6)] (Table S1, and Figure 1).

#### *COMT val158met* genotype

Subjects who were carriers of met/* alleles showed a higher mean MDMA K_e_ compared to *val/val* individuals [*p* = 0.006, 95% CI = 0.02–0.09] (Table S1).

### Physiological Effects

MDMA produced the well-known physiological and subjective effects described in the literature [29], [21]. It increased BP, HR, OT, PD, ESO and induced euphoria, stimulation, and well-being. None of participants required specific therapy or special care due to adverse events.

#### Gender

**Figure 2:**
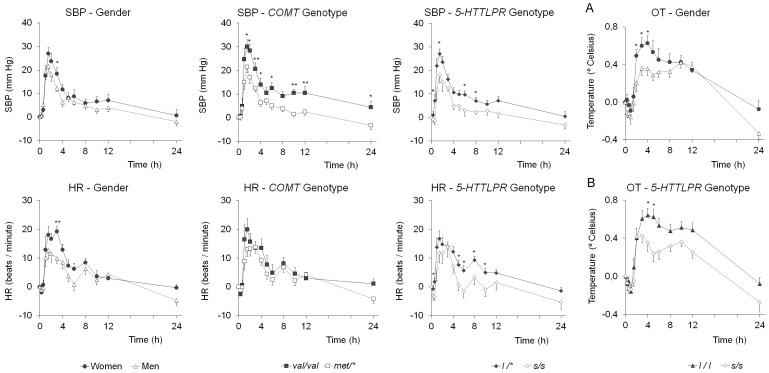
Influence of gender and genetics (*COMT, 5-HTTLPR*) on the temporal course of systolic blood pressure (upper-left panel), heart rate (lower-left panel), and oral temperature (right-end panel) (mean ± SEM); women n = 12 *vs.* men n = 15; COMT, *val/val* n = 8 vs. met/* n = 18; 5-HTTLPR, l/* n = 18 *vs. s/s* n = 9). *p<0.05, **p<0.01. Graph A corresponds to gender differences in OT, graph B corresponds to differences in OT as a function of 5-HTTLPR polymorphisms (*l/l* n = 11 *vs. s/s* n = 9). Subjects *l/s* (n = 7) are not represented for graph clarity, but data almost fully overlaps with the *s/s* trace.

Women presented higher values than men in HR [AUC _0–4__h_ (*p* = 0.009)_,_ AUC_0–6__h_ (*p = *0.013) and E_max_ (*p* = 0.001)]; and OT [AUC _0–4__h_ (*p* = 0.038) and AUC_0–6__h_ (*p = *0.017)] (Table 4 and Figure 2). Men showed a trend towards significance in PD [AUC_0–24__h_ (p = 0.057)].

#### *COMT val158met* genotype

The *val/val* allele carriers presented higher values of SBP [AUC_0–4__h_ (*p* = 0.012), AUC_0–6__h_ (*p* = 0.013), AUC_0–24__h_ (*p = *0.005) and E_max_ (*p = *0.037,)] and DBP [AUC_0–24__h_ (*p = *0.038)] compared to met/* ones (Table 5 and Figure 2).

#### *5-HTTLPR* genotype

The l/* allele carriers presented higher values of SBP [AUC_0–4__h_ (*p* = 0.004), AUC_0–6__h_ (*p* = 0.004), AUC_0–24__h_ (*p = *0.022) and E_max_ (*p = *0.002)]; DBP [E_max_ (*p* = 0.009)]; and HR [AUC_0–6__h_ (*p* = 0.017), AUC_0–24__h_ (*p* = 0.018*),* with a trend towards significance in E_max_ (*p* = 0.052)] than *s/s* carriers (Table 5 and Figure 2).

### Subjective Effects

#### Gender

Women displayed higher scores than men in VAS–Dizziness [AUC_0–4__h_ (*p* = 0.006) and AUC_0–6__h_ (*p* = 0.005), AUC_0–24__h_ (*p* = 0.016), and E_max_ (*p* = 0.029)]; VAS–Depression or Sadness [AUC_0–6__h_ (*p* = 0.041) and E_max_ (*p* = 0.047)]; ARCI–PCAG Group (sedation) [AUC_0–24__h_ (*p* = 0.011) and E_max_ (*p* = 0.042)]; VESSPA–Sedation [AUC_0–4__h_ (*p* = 0.047), AUC_0–6__h_ (*p* = 0.028), AUC_0–24__h_ (*p* = 0.005) and E_max_ (*p* = 0.002] (Table 4); and VESSPA-Psychotic symptoms [AUC_0–24__h_ (*p* = 0.048)].

#### *COMT val158met* genotype

The met/* allele carriers presented higher response in VAS-Dizziness [AUC_0–4__h_ (*p* = 0.014), AUC_0–6__h_ (*p* = 0.020), AUC_0–24__h_ (*p = *0.031) and E_max_ (*p = *0.022)], and VESSPA-ANX [AUC_0–24__h_ (*p = *0.025)] than *val/val* carriers (Table 5).

#### *5-HTTLPR* genotype

The *s/s* allele carriers presented higher values of VESSPA-Sedation [AUC_0–4__h_ (*p* = 0.045)] in relation to subjects with l/* alleles (Table 5).

No other differences according to gender and genetics were observed in scales not previously discussed.

## Discussion

To our knowledge, this is the first study on the human pharmacology of MDMA that reports differences in pharmacokinetics and physiological-subjective effects, taking into account gender and genetics, after the administration of doses compatible with its recreational consumption. We have confirmed that there are marked gender differences in MDMA pharmacology: women experienced heightened physiological effects, in particular, cardiovascular ones. They also reported greater negative subjective effects (e.g. dizziness, depression/sadness, and sedation). These observations were not related to MDMA or MDA pharmacokinetics as no major differences in their metabolic disposition at the dose studied were reported between genders or studied genotypes. In the studied population of extensive metabolizers the *CYP2D6* genotype was only relevant in the plasmatic concentrations of HMMA depending on the carried number of functional alleles. Furthermore, gender and *COMT val158met* and *5-HTTLPR* polymorphisms played a major role in the physiological and subjective MDMA induced effects. Genotypes corresponding to protein high functionality (e.g. *5-HTTLPR* l/* genotype or *COMTval158met* val/val genotype) were associated with heightened cardiovascular effects.

### Pharmacokinetics

The main findings are that women, when they received similar weight-adjusted doses as men (1.4 mg/kg, but at a significantly lower total dose, 80 mg *vs*. 95 mg), reached similar plasma concentrations of MDMA and metabolites.

Our results partially differ from those of Kolbrich *et al* [10] where a multiethnic group of 17 subjects (6 females) received low (1.0 mg/kg) and high (1.6 mg/kg) oral MDMA doses in a double-blind, randomized, placebo-controlled study. While our results are similar to those reported by Kolbrich *et al* [10] at the high dose as no gender differences were observed; in contrast, gender differences were observed with the lower dose. This may be due to the fact that at lower MDMA doses the impact of CYP2D6 autoinhibition in its metabolic disposition is less relevant than at the higher ones. Secondly, discrepancies observed between our study and Kolbrich´s work could be due to differences in the power of the sample size (n = 27 *vs.* n = 17), genetic polymorphisms of *CYP2D6, COMT,* and *5-HTT* (not measured in Kolbrich’s study), and ethnicity that might have influenced the activity of these proteins [31]–[33].

#### MDMA

Similar plasma concentrations in men and women are consistent with Kolbrich´s results at high dose [10]. Furthermore, our results in K_a_ and Vd are also in agreement with the literature [18], [10]. From a metabolic point of view, although oral doses assayed reached the same level of CYP2D6 inhibition, men 80% and women 86% (*p* = 0.15) [18], the slightly higher MDMA concentrations in women are most probably related to a lower CYP2D6 baseline activity than men [22], and to a lower plasmatic MDMA Cl.

According to the elimination parameters, men presented a significantly higher MDMA Cl than women, but no gender differences were observed in relation to K_e_ and T_1/2_. Variations in Cl, although not determinant to generate gender differences in MDMA plasma concentrations, could be due to the fact that the Cl of drugs is generally higher in men than in women (25%) [34], [22], [10]. In addition, the higher baseline CYP2D6 activity in men would imply, despite the autoinhibition of the enzyme, a greater capacity to clear the drug [22].

#### HMMA

Our results show that HMMA formation is conditioned more by *CYP2D6* polymorphism than by COMT val158met. A higher CYP2D6 enzymatic activity (carriers of 2 FA) is determinant in HMMA plasma concentrations. In contrast, the different enzymatic activity of *COMT val158met* genotype may partially explain the inter-individual variability in susceptibility to MDMA-induced neurotoxicity [17], [20], [35]. Finally, gender does not appear to be relevant in HMMA formation, although men tend to present greater concentrations because of the higher activities of CYP2D6 and COMT [22], [36], as reflected in the lower MDMA vs. HMMA AUC_0-12__h_ ratio. CYP2D6 and COMT genotypes have been recently associated with lower sodium in plasma and/or higher cortisol levels among MDMA users [37], [38].

#### MDA

Similar plasma concentrations in men and women are consistent with Kolbrich´s results at the 1.6 mg/kg dose [10]. Slightly higher concentrations of MDA observed in women (Table 3) could be due to the lower CYP2D6 activity that implies both a higher availability of substrate (MDMA) for N-demethylation to MDA and a simple accumulation of MDA, since the O-demethylenation pathway from MDA to HHA is also regulated by CYP2D6 [21].

### Physiological Effects

In the absence of pharmacokinetic differences between the genders, the most important findings in our study were that female gender and the *5-HTTLPR* and the *COMT val158met* polymorphisms play a major role in modulating the physiological and subjective effects of MDMA.

The main results show that women presented more intense effects in SBP, HR, and OT than men, while subjects with high functionality in *5-HTTLPR* (l/* alleles) or *COMT val158met* (*val/val* alleles) genotypes experienced increased cardiovascular effects.

The MDMA-induced cardiovascular effects are mainly due to the release of NE and 5-HT [39], [40]. Gender differences in the 5-HT neurotransmission system, which is modulated by sex steroids, could have contributed to our results. Women exhibited an enhanced serotonergic function in relation to men [15], [41]. On the other hand, men have larger reserves of 5-HT, and, therefore, more extensive disturbances in 5-HT synthesis/transmission may be required to respond to MDMA [42].

Genetics determined blood pressure (BP) and HR effects. Our results are plausible since MDMA promotes 5-HT release through the translocation of 5-HTT and, as a result, the availability of 5-HT in the synapse will be greater in the l/* carriers because they present higher expression of 5-HTT in relation to the *s*/s carriers. Furthermore, results of BP according to *COMT val158met* genotype are consistent with the literature [43], and independent of BP results regarding *5-HTTLPR* genotype since the proportion of subjects with l/* and *s/s* alleles in *val/val* and met/* groups was similar.

MDMA-induced effects on HR are determined by female gender and *5-HTTLPR* genotype. However, gender might be more important than genetics; in fact, women showed higher HR increases than men in all four subgroups of genotypes (no differences in HR increase were observed between women carrying l/* and *s/s* allelic-variants, results not shown). The results on HR are clinically relevant because the mean 20 beats/min (bpm) HR increase developed into tachycardia in some cases. Furthermore, HR increase would be greater in a real context of drug consumption than that observed in our laboratory controlled study because consumers dance for hours under stressful conditions (loud music and high ambient temperature), and repeated doses may be taken per session. Globally, this would worsen observations made in the experimental setting. Naturalistic studies suggest that the HR of ecstasy users easily reaches 100 bpm. [44], [45]. Values that are comparable with those observed during physical exercise of variable intensity in healthy people [46], [47].

The modest increase observed in OT, which is well-documented, seems to be influenced by gender, although the contribution of the *5-HTTLPR* polymorphism should not be completely discarded [5], [48], [30].The sexual dimorphism in the 5-HT system (previously discussed) may explain gender differences. When comparing OT values of *l/l* vs. *s/s* carriers there were differences in the AUC_0-8__h_ (*p* = 0.015) (Figure 2). The increase of OT observed between 8–12 h post-administration of MDMA could be simply physiological (circadian rhythm).

In the work of Liechti *et al* [8] whilst no gender differences were reported in DBP, HR, and peripheral body temperature they were, however, observed in SBP where men displayed significantly higher values. Women showed a significant increase in SBP and DBP compared to placebo. Discrepant results between our study and Lietchís could be due to the fact that the volunteers of Liechti and colleagues were drug naïve, received similar or higher MDMA doses (1.35–1.8 mg/kg) than ours, and genetic polymorphisms were not considered.

### Subjective Effects

The main findings are that some negative effects were modulated by female gender and *5-HTTLPR* or *COMT val158met* genotypes whereas similar positive effects were observed irrespective of gender or genotypes.

Women experienced significant effects in dizziness, depression/sadness and psychotic symptoms, and sedation all of which are consistent with the literature [45], [5], [10], [21]. The negative subjective effects observed in women could be due to a higher predisposition to suffer from psychological/mood disorders than men [49], [15]. MDMA may act as a trigger for such symptoms by the depletion of the limited 5-HT reserves in women. In contrast, men could compensate for the psychological adverse effects of MDMA through faster synthesis and larger reserves of 5-HT [50].

The *5-HTTLPR* and *COMT val158met* genotypes with low functionality also influenced subjective effects. Sedation was determined by *s/s* alleles whilst dizziness and anxiety depended on met/* alleles [15]. A larger number of adverse effects in met/* carriers have been reported after amphetamine administration [51].

No gender differences were observed in positive subjective effects as reported by other authors of psychostimulant studies [52], [45], although some have suggested that this may depend on the phase of the menstrual cycle [53]. Nevertheless, as far as MDMA effects are influenced by environment (e.g. increased in a rave party), gender differences may exist, and the neutral environment of this experiment should also be considered [45].

A major limitation of the present study was that it was relatively small considering the large number of statistical tests that were not corrected for multiple comparisons. Consequently, a larger study is needed to confirm the findings. The lack of statistical power might have limited the detection of minor gender differences in MDMA plasma concentrations. Nonetheless, drugs×gender interaction findings are independent of *CYP2D6, COMT,* or *5-HTTLPR* genotype distributions among males and females. Moreover, we should point out that our drugs×gene interaction findings are in agreement with initial assumptions, biologically plausible, and consistent with the previous literature. Therefore, although these results need to be further explored and replicated, the probability that they stem from false positive effects is low. Outlying values were distributed in a similar way between genders and genotypes and did not contribute to differences observed among experimental groups. We are aware that our results concern mainly extensive and intermediate metabolizers of CYP2D6 which represent about 90% of the general population. Extreme phenotypes (poor and ultrarapid metabolizers) were not included because in the screening of more than sixty-five subjects, recreational users of MDMA, genotyped for CYP2D6, these phenotypes were underrepresented. Two poor metabolizers were not considered for their inclusion due to comorbid psychopathology, and none of the subjects carried duplications of CYP2D6 functional alleles leading to the ultrarapid phenotype. Similar findings have been reported in other series of MDMA users [38]. Apparently, there are fewer subjects with these extreme phenotypes who consume MDMA in comparison with the general population. As reported earlier, poor metabolizer subjects may display increased plasma concentrations and more intense pharmacological effects after a single dose of MDMA [12], similar to those experienced by extensive metabolizers after two consecutive doses, because of a phenomenon of phenocopying towards the poor metabolizer phenotype due to CYP2D6 autoinhibition. A larger and more comprehensive study is needed to more conclusively evaluate the effect of CYP2D6 function on the effects of MDMA. In particular, it needs to be examined whether CYP2D6 poor metabolizers exhibit increased exposure to MDMA and an enhanced pharmacodynamic response to the drug.

The large number of statistical tests applied was not corrected. Although this attitude implies a strong probability of a Type 1 error, lowering the significance level (for example to 0.01) would have implied an even greater increase of the probability of a Type 2 error because of the small sample size.

At the dose administered (1.4 mg/kg) no major gender pharmacokinetic differences were reported. They could, however, be observed with lower (1 mg/kg) [10] doses of MDMA than those assayed.

In conclusion, there are marked gender differences in MDMA pharmacology. Women experience greater heightened physiological and negative subjective effects than men. This observation does not appear to be related to gender differences in drug disposition at the dose assayed of 1.4 mg/kg. The *5-HTTLPR* and/or *COMT val158met* polymorphisms combine to play an important role in modulating risk for MDMA adverse effects, mainly cardiovascular ones.

## Supporting Information

Table S1 **Genetic (** ***CYP2D6, COMT*** **) differences in pharmacokinetic parameters of MDMA and its metabolites (mean ± SD).**
(DOCX)
