# Supplementary material for: Clinical Pharmacology of 3,4-Methylenedioxymethamphetamine (MDMA, “Ecstasy”): The Influence of Gender and Genetics (CYP2D6, COMT, 5-HTT)
Source: PLoS One. 2012 Oct 24;7(10):e47599. doi: 10.1371/journal.pone.0047599 (PMC3480420; doi:10.1371/journal.pone.0047599)
Supplement: Table S1 — Genetic ( CYP2D6, COMT ) differences in pharmacokinetic parameters of MDMA and its metabolites (mean ± SD). (DOCX) [file pone.0047599.s001.docx]

**Table S3.**

|  | **AUC** _0-25h_ (µg·h·L^-1^) | **C_max_** (µg/L) | **T_max_**(h) | **T _1/2_** (h) | **K_e_** (h^-1^) |
| --- | --- | --- | --- | --- | --- |
| **MDMA** |  |  |  |  |  |
| *2 AF* | 2054.0 ± 374.6 | 196.3 ± 46.1 | 2.9 ± 1.3 | 8.4 ± 7.7 | 0.1 ± 0.04 |
| *1 AF* | 2029.1 ± 673.4 | 171.5 ± 51.6 | 2.2 ± 0.7 | 14.7 ± 16.7 | 0.08 ± 0.05 |
| *val/val* | 2287.1 ± 417.0 | 179.4 ± 41.8 | 2.7 ± 1.5 | 15.3 ± 16.2 | 0.07 ± 0.02 |
| *met/** | 1939.3 ± 468.8 | 192.8 ± 51.8 | 2.7 ± 1.0 | 8.1 ± 16.3 | 0.12 ± 0.05****** |
| **HMMA** |  |  |  |  |  |
| *2 AF* | 2408.6 ± 1034.4****** | 219.3 ± 95.3****** | 2.7 ± 1.6 | 11.2 ± 3.9 | 0.07 ± 0.03 |
| *1 AF* | 1105.6 ± 614.9 | 97.05 ± 53.71 | 3.7 ± 2.7 | 14.5 ± 14.4 | 0.2 ± 0.28 |
| *val/val* | 1750.6 ± 1286.5 | 158.3 ± 127.8 | 3.4 ± 2.8 | 16.8 ± 11.2 | 0.05 ± 0.02 |
| *met/** | 2121.9 ± 1074.3 | 192.1 ± 98.3 | 2.9 ± 2.6 | 10.2 ± 11.9 | 0.14 ± 0.18 |
| **MDA** |  |  |  |  |  |
| *2 AF* | 206.1 ± 39.1 | 14.07 ± 3.9 | 6.9 ± 2.8 | 16.5 ± 8.7 | 0.05 ± 0.02 |
| *1 AF* | 206.3 ± 46.1 | 12.5 ± 1.9 | 7.1 ± 3.4 | 23.0 ± 11.8 | 0.04 ± 0.01 |
| *val/val* | 206.7 ± 38.5 | 14.4 ± 3.6 | 8.0 ± 3.3 | 20.2 ± 11.1 | 0.04 ± 0.02 |
| *met/** | 205.9 ± 40.8 | 13.2 ± 3.3 | 6.5 ± 3.0 | 17.5 ± 12.4 | 0.05 ± 0.02 |
| **HMA** |  |  |  |  |  |
| *2 AF* | 103.4 ± 50.3 | 6.6 ± 3.0 | 7.5 ± 3.0 | n.d | n.d |
| *1 AF* | 75.2 ± 25.1 | 4.7 ± 1.5 | 8.6 ± 3.2 | n.d | n.d |
| *val/val* | 81.6 ± 38.8 | 5.8 ± 3.3 | 6.5 ± 3.0 | n.d | n.d |
| *met/** | 103.7 ± 48.5 | 6.2 ± 2.5 | 8.5 ± 3.0 | n.d | n.d |

AUC: area under the concentration-time curve, C_max_: peak plasma concentration; T_max_: time with peak plasma; T _1/2_: half-life of elimination; K_e_: elimination constant. FA: number of functional alleles. N.d: not determined due to high variability. ** *p* <0.01. Subjects with 2 FA n=18 *vs*. with 1 FA n=8. Subjects with *val/val* alleles n=8, *vs*. with *met/** alleles n=18. MDMA, ±3,4-methylenedioxymethamphetamine; MDA, 3,4-methylenedioxyamphetamine; HMA, 3-methoxy-4-hydroxyamphetamine; HMMA, 3-methoxy-4-hydroxymethamphetamine.
